# In the era of targeted therapy and immunotherapy: advances in the treatment of large B-cell lymphoma of immune-privileged sites

**DOI:** 10.3389/fimmu.2025.1547377

**Published:** 2025-04-01

**Authors:** Haotian Wang, Ying Zhang, Xin Wan, Zhaoxia Li, Ou Bai

**Affiliations:** Department of Hematology, The First Hospital of Jilin University, ChangChun, Jilin, China

**Keywords:** primary large B-cell lymphomas of immune-privileged sites, targeted therapy, immunotherapy, efficacy, safety

## Abstract

Primary large B-cell lymphomas of immune-privileged sites (IP-LBCLs) include primary central nervous system large B-cell lymphoma (PCNSL), primary vitreoretinal large B-cell lymphoma (PVRL), and primary testicular large B-cell lymphoma (PTL). These tumors not only have a unique anatomical distribution but also exhibit specific biological and clinical characteristics. Given the high biological overlap between intravascular large B-cell lymphoma (IVLBCL) and IP-LBCLs, and the fact that IVLBCL is confined to the intravascular microenvironment, IVLBCL is currently included in the category of IP-LBCLs. IP-LBCLs are associated with suboptimal prognosis. However, advancements in biomarker detection technologies have facilitated novel therapeutic approaches for this disease entity. This review aims to summarize and analyze the latest research progress in IP-LBCLs, with a focus on new treatment strategies in the era of targeted therapy and immunotherapy. It is intended to further understand the biological characteristics, treatment, and latest advancements of this disease.

## 1 Introduction

Primary large B-cell lymphomas of immune-privileged sites (IP-LBCLs) are classified as an independent entity in the 5th edition of the World Health Organization (WHO) classification of lymphoid neoplasms (1). These lymphomas occur specifically in immunocompetent individuals, within specific anatomic sites behind immune barriers such as the blood-brain barrier (BBB), blood-retinal barrier, and blood-testicular barrier, as well as within the immunoregulatory systems that form immune sanctuaries in these primary sites (2). IP-LBCLs include primary central nervous system large B-cell lymphoma (PCNSL), primary vitreoretinal large B-cell lymphoma (PVRL), and primary testicular large B-cell lymphoma (PTL) (1, 2). Lymphomas arising at other sites, such as intravascular large B-cell lymphoma (IVLBCL), also appear to share similar characteristics (2). In IP-LBCLs, PCNSL is the most common, accounting for 4-6% of all extranodal lymphomas (3, 4). The clinical presentation is most commonly characterized by focal neurologic deficits, such as fatigue, sensory changes, and aphasia (4). Compared with other extranodal large B-cell lymphomas, PCNSL has a poorer prognosis with a 5-year overall survival (OS) of only 30% (5–7). Despite the fact that high-dose methotrexate (HD-MTX)-based combination chemotherapy is the first-line treatment for PCNSL, there is a recurrence rate of up to 50% in patients (8–10). Recurrent or refractory PCNSL (R/R PCNSL) has an extremely poor prognosis, with no standard treatment protocol. PVRL is a rare subtype of PCNSL that originates in the vitreoretinal area without CNS involvement (11). The clinical presentation is mostly localized ocular symptoms, such as blurred vision, decreased visual acuity, and floaters. Unlike PCNSL, PVRL can be treated locally, such as by intraocular injection of chemotherapy drugs or ocular radiotherapy (8, 12–15). PTL accounts for 1-2% of non-Hodgkin’s lymphomas (NHL) and is the most common malignant testicular tumor in men over 60 (16–18). PTL is usually confined to the testicle but can also spread throughout the body, such as to the CNS, skin, lung, soft tissue, and contralateral testicle. The majority of patients present with painless testicular enlargement, and up to 40% of cases also have scrotal hydrocele. Surgery is the first-line treatment for suspected PTL. IVLBCL is a rare subtype of NHL characterized by a large number of tumor cells aggregating in small or medium-sized vascular lumens, particularly capillaries and postcapillary venules. It is prone to invasion of the CNS but rarely involves lymph nodes (19). IVLBCL is divided into three subtypes, namely the classic subtype, skin subtype, and hemophagocytic-related subtype, with different clinical presentations among the subtypes. However, the overall prognosis is extremely poor, with a median survival of approximately 1 year (20)(**Table 1**).

**Table 1:** Characteristics of IP-LBCLs.

	PCNSL	PRVL	PTL	IVLBCL
Site of Origin	Brain, spinal cord, leptomeninges, or vitreoretinal space	Vitreous, retina, without CNS involvement	Mostly confined to the testis	Small or medium-sized vascular lumens
Incidence	0.3-0.6 per 100,000; 4-6% of extranodal lymphomas; 3% of CNS tumors	1% of ocular malignancies	0.09-0.26 per 100,000; 1-2% of NHL; 5% of testicular malignancies	0.5 per million
Median age	60-65 years	50-70 years	66-68 years	Around 70 years
Clinical presentation				Classic subtype: Fever, pain, specific symptoms of local organs, or multiple organ failure.
	Focal neurological deficits; Neuropsychiatric and behavioral changes	Floaters, blurred vision, decreased vision	Painless testicular enlargement, hydrocele in 40% of cases	Skin subtype: Skin lesion changes.
				Hemophagocytic subtype: Multi-organ failure; Hepatosplenomegaly; Pancytopenia.
Survival	5-year OS: 30%	Median PFS: 18-29 months; median OS: 58-75 months	5-year OS: 87%	Median survival: 1 year
Treatment Strategies	Surgical resection; Radiotherapy; Chemotherapy; Immunotherapy	Intraocular chemotherapy; Ocular radiotherapy; Systemic treatment (refer to PCNSL)	Surgical resection; Radiotherapy; Chemotherapy; Immunotherapy	Immunotherapy

IP-LBCLs, Primary Large B-cell Lymphomas of Immune-Privileged Sites; PCNSL, primary central nervous system lymphoma; PVRL, primary vitreoretinal lymphoma; PTL, primary testicular lymphoma; IVLBCL, intravascular large B-cell lymphoma; NHL, non-Hodgkin lymphomas; PFS, progression-free survival; OS, overall survival.

**Figure 1:**
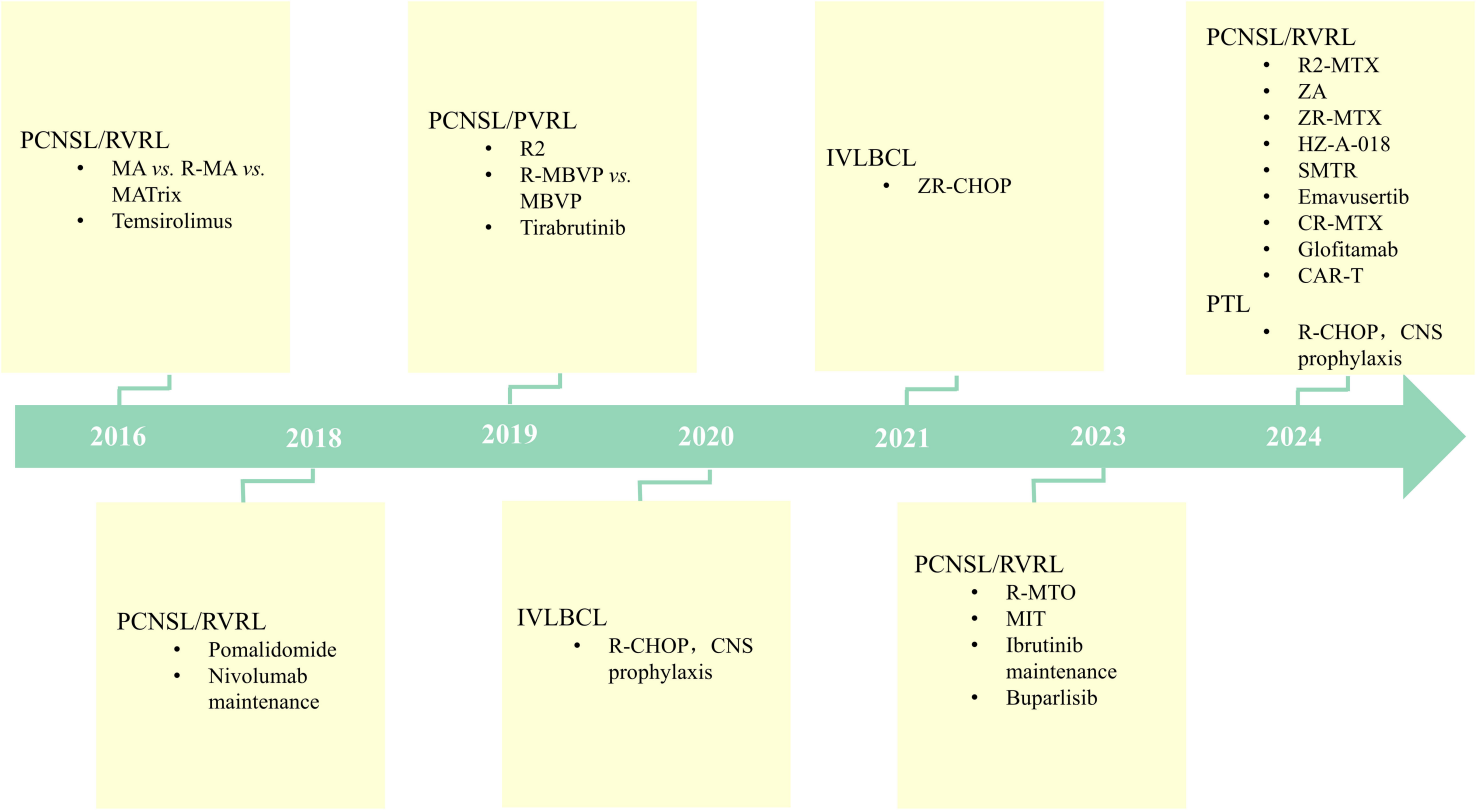
Therapeutic Advances in IP-LBCLs: Targeting and Immunotherapy in the Modern Era. (IP-LBCLs: primary large B-cell lymphomas of immune-privileged sites; PCNSL, primary central nervous system lymphoma; PVRL, primary vitreoretinal lymphoma; PTL, primary testicular lymphoma; IVLBCL, intravascular large B-cell lymphoma; R-MBVP, rituximab/methotrexate/carmustine/teniposide/prednisone; R-MA, rituximab/methotrexate/cytarabine; MARix, methotrexate/cytarabine/rituximab/thioepa; R-CHOP, rituximab/cyclophosphamide/doxorubicin/vincristine/prednisone; MIT, methotrexate/ibrutinib/temozolomide; ZA, zanubrutinib/cytarabine; ZR-MTX, zanubrutinib/rituximab/methotrexate; CR-MTX, chidamide/rituximab/methotrexate; R-MTO, methotrexate/orelabrutinib/thiotepa/rituximab; R2, lenalidomide/rituximab; POM, pomalidomide; SMTR, sintilimab/methotrexate/temozolomide/rituximab; CAR, chimeric antigen receptor).

IP-LBCLs are associated with a poor prognosis and currently lack standardized treatment regimens. Molecular characterization reveals distinct heterogeneity in IP-LBCLs, primarily manifesting through the following features: High-frequency genetic mutations (2): MYD88 mutations drive constitutive activation of the NF-κB signaling pathway, promoting tumor cell proliferation and survival. CD79B mutations amplify BCR signaling, synergizing with MYD88 mutations to enhance NF-κB pathway activation. Genomic instability (21): Frequent 9p24.1/PD-L1/PD-L2 copy number alterations. Increased PD-L1/PD-L2 copy numbers may underlie immune evasion mechanisms in IP-LBCLs. These molecular hallmarks have catalyzed the development of novel targeted therapies and immunotherapeutic strategies, offering promising alternatives for IP-LBCL management (**Figure 1**).

## 2 Current treatment strategies

### 2.1 Targeted therapy

#### 2.1.1 Bruton tyrosine kinase inhibitors

In normal B cells, the Toll-like receptor (TLR) signaling pathway and the B-cell receptor (BCR) signaling pathway synergistically activate the NF-κB signaling pathway. However, when MYD88 and CD79B mutations occur, they abnormally activate the NF-κB signaling pathway, thereby promoting malignant proliferation of B cells, which is an important mechanism in the formation of B-cell lymphoma. IP-LBCLs exhibit similar phenotypes and molecular characteristics, with most belonging to the MCD subtype, harboring mutations in MYD88 and CD79B (2). Notably, more than 60% of IP-LBCLs patients have MYD88 mutations, with mutation rates reaching up to 88% in subtypes such as PVRL, 67% in PCNSL, between 68-82% in PTL, and about 60% in IVLBCL (11, 22–24). In systemic DLBCL, molecular subtyping based on next-generation sequencing has stratified the disease into four distinct genetic classifications: EZB (EZH2 mutations and BCL2 translocations), MCD (MYD88 and CD79B mutations), BN2 (BCL6 translocations and NOTCH2 mutations), and N1 (NOTCH1 mutations) subtypes. Notably, both MCD and N1 subtypes exhibit inferior prognosis, with the MCD cohort demonstrating a particularly dismal 5-year OS rate of 40% (25, 26). This molecular heterogeneity likely contributes to the poor clinical outcomes observed in IP-LBCLs. MYD88, TLR9, and BCR form the “My-T-BCR” supercomplex, which is the activation site of NF-κB. More significantly, this site is highly sensitive to BTKi (27, 28). Moreover, as a small molecule inhibitor, BTKi can effectively cross the BBB (29–31). Therefore, the application of BTKi in IP-LBCLs treatment has become a hot topic in current research.

In the phase I/II study of ibrutinib monotherapy in R/R PCNSL (32), an objective response rate (ORR) of up to 77% was observed regardless of BCR and MYD88 status. In a phase II study involving 46 patients with R/R CNSL (33), the ORR of monotherapy ibrutinib in PCNSL patients reached 74%. Moreover, in a multi-center, prospective phase II study (34) using the MIT regimen (HD-MTX + ibrutinib + temozolomide) for the initial treatment of PCNSL, a total of 33 patients were included. After receiving MIT induction therapy, they were given either sequential autologous stem cell transplantation (ASCT) consolidation treatment or ibrutinib maintenance treatment. The best ORR was 94%, with a median follow-up of 17.5 months, with a 1-year progression-free survival (PFS) and OS rate of 66.4% and 90.5%, respectively, and no severe adverse events (SAEs)-related deaths were observed during treatment. In another study from China (35), the ZR-MTX regimen (zanubrutinib+rituximab+HD-MTX) was used to treat newly diagnosed PCNSL in 15 patients, with an ORR of 66.7% and a complete response rate (CRR) of 40.0%. With a median follow-up of 20.2 months, the 2-year PFS and OS were 38.9% and 67.5%, respectively. Notably, no unexpected toxicities were observed throughout the treatment process. In a phase II prospective study by Bairey et al. (36), ibrutinib monotherapy was used as a maintenance treatment for newly diagnosed PCNSL after HD-MTX-based induction therapy. A total of 20 patients were included, and the median duration of ibrutinib maintenance was 12.5 months. The results showed a 2-year PFS and OS of 72.6% and 89%, respectively, with most AEs being of grade 1/2. In another ongoing single-center, single-arm, open-label study (37), 19 patients with newly diagnosed PCNSL were given 5-8 cycles of R-MTO (rituximab+HD-MTX+thiotepa+orelabrutinib) followed by sequential ASCT consolidation treatment. After 4 cycles of induction therapy, the CRR reached 89.47%, and the most common AE related to the R-MTO regimen were grade 1-2 neutropenia.

Tirabrutinib, as a second-generation BTKi, exhibits higher selectivity than ibrutinib. In a phase I/II study of tirabrutinib monotherapy for R/R PCNSL (38), a total of 44 patients with R/R PCNSL were enrolled, receiving single-agent tirabrutinib oral administration at different dosages. The ORR was 64%, with a median PFS of 2.9 months. Among these, 1 patient was observed to have a grade 5 AE. HZ-A-018 is a novel, highly selective covalent BTK inhibitor, which also possesses the ability to cross the BBB. The ongoing HZ-A-018-102 study is a phase I/II clinical trial (39), designed to assess the efficacy and safety of HZ-A-018 in CNS lymphoma. A total of 29 patients with R/R CNSL were enrolled, including 26 with PCNSL. As of the latest data analysis, with a median follow-up of 19 months, the ORR was 50%, and the median OS had not been reached. In terms of safety, only 5 patients experienced treatment-related AEs(TRAEs) of grade ≥3, and none of the patients had their dosages reduced or treatment discontinued due to TRAEs. Further exploratory studies have shown that the combination of HZ-A-018 with HD-MTX demonstrates preliminary efficacy in patients with R/R and newly diagnosed PCNSL, with an ORR of 55.6% among 9 R/R PCNSL patients (40). In 10 patients with newly diagnosed PCNSL, 2 have completed induction therapy and achieved a PR.

A prospective, single-arm, phase II study (41) conducted at Peking Union Medical College Hospital in China included 9 patients with IVLBCL, who received zanubrutinib combined with R-CHOP (rituximab + cyclophosphamide + doxorubicin + vincristine + prednisone) for 8 cycles, achieving an ORR of 100%. At a median follow-up of 10 months, there was no recurrence observed in any of the patients.

In summary, the application of BTKi in IP-LBCLs has undoubtedly been successful, regardless of whether it is used for newly diagnosed or relapsed/refractory patients, whether as induction or maintenance therapy, it has yielded extremely impressive results.

### 2.2 Immunotherapy

#### 2.2.1 Anti-CD20 monoclonal antibody

##### 2.2.1.1 Rituximab

RTX is a human-mouse chimeric monoclonal antibody that primarily targets the CD20 antigen on the surface of B cells. Binding of this antibody to the CD20 antigen effectively blocks the normal function of B cells and rapidly triggers their depletion (42, 43). Upon binding to the CD20 antigen on the surface of B cells, RTX activates the complement system, leading to the lysis of B cells through complement-dependent cytotoxicity (CDC). Concurrently, macrophages and natural killer cells (NK cells) can bind to the Fc portion of RTX, mediating antibody-dependent cellular cytotoxicity (ADCC), which further clears CD20-positive B cells (44, 45).

Due to the large molecular weight of RTX, uncertainties persist regarding its ability to accumulate in the CNS at therapeutically effective concentrations required for anti-lymphoma activity. Pharmacokinetic studies demonstrate that intravenous RTX administration achieves cerebrospinal fluid concentrations <0.1% of serum levels, insufficient for sustained target engagement in CNS compartments (46). Notably, Lwamoto et al (47). demonstrated that radiation-induced BBB disruption via stereotactic radiosurgery significantly enhanced RTX penetration into the CNS parenchyma, thereby achieving therapeutic concentrations. These data highlight the critical dependency of RTX efficacy on BBB permeability modulation, contributing to ongoing controversies in its clinical utility for PCNSL management.

Several key clinical studies currently provide important evidence for clinical practice. Among them, the HOVON 105/ALLG study (48) is an open-label, randomized, controlled, multicenter phase III trial that enrolled 200 patients with normal immune function who had been newly diagnosed with PCNSL. These patients were randomly assigned to two groups in a 1:1 ratio, receiving either MBVP (HD-MTX+ carmustine + teniposide + prednisone) or R-MBVP induction therapy. With a median follow-up of 32.9 months, there were no significant differences between the two groups in terms of event-free survival (EFS) (HR=1.00, 95% CI: 0.70-1.43, P=0.99), PFS (HR=0.77, 95% CI: 0.52-1.13, P=0.18), and OS (HR=0.93, 95% CI: 0.59-1.44, P=0.74). Another study, IELSG32 (49, 50) is a multicenter, randomized, phase II trial that included a total of 227 patients with newly diagnosed PCNSL. These patients were randomly divided into three groups and received different induction chemotherapy regimens: (A) HD-MTX + cytarabine; (B) HD-MTX + cytarabine + RTX; (C) HD-MTX + cytarabine + RTX + thiotepa. With a median follow-up of 88 months, the results showed that the PFS of group B was slightly better than that of group A, although there was no significant statistical difference (P=0.06), but group B significantly prolonged the OS (P=0.04). In addition, a 1:1 propensity-matched study based on the National Cancer Database (51) found that compared with conventional chemotherapy, the combination of RTX immunotherapy significantly improved the OS of PCNSL patients (HR=0.75, 95% CI: 0.67-0.83). A meta-analysis (52) demonstrated improved PFS with RTX-containing regimens in PCNSL patients, while failing to establish significant OS benefit.

##### 2.2.1.2 Combined chemotherapy and immunotherapy

In the treatment of PTL, the immunotherapy regimen R-CHOP, has become the mainstream treatment option. Recent research data show that the 5-year OS rate for patients treated with the R-CHOP regimen is 86.6% (53). In addition, the prospective results from studies IELSG-10 and IELSG-30 further confirm the advantages of the R-CHOP regimen in the treatment of PTL (54, 55). These studies used the R-CHOP regimen as induction therapy and combined it with radiotherapy and intensive CNS prophylaxis. This comprehensive treatment approach can effectively reduce the risk of CNS recurrence, thereby further improving the overall efficacy of PTL treatment. In the treatment of IVLBCL, the PRIMEUR-IVL study is a significant multicenter, single-arm phase II clinical trial (56). This study included 37 patients with *de novo* IVLBCL who received immunotherapy based on RTX, combined with CNS prophylactic treatment. With a median follow-up of 3.9 years, the results showed that the 2-year PFS and OS rates were 76% and 92%, respectively. The latest 7-year follow-up data demonstrated 5-year PFS and OS rates of 68% and 78% respectively, with a cumulative CNS event incidence of 3% (57).

On the whole, despite the fact that there is no unified consensus on the application of RTX in PCNSL treatment, immunotherapy based on RTX has demonstrated significant efficacy in the treatment of PTL and IVLBCL, providing clinicians with an effective therapeutic approach.

#### 2.2.2 Immunomodulatory agents

As a new strategy in immune therapy, IMiDs have demonstrated significant anti-tumor potential. These drugs exert multiple effects such as regulating the tumor microenvironment, inhibiting angiogenesis, enhancing immune surveillance, modulating cell signaling pathways with immunological activity, and promoting tumor cell apoptosis (58). IMiDs, exemplified by lenalidomide (Len), exert antitumor effects through multifaceted molecular mechanisms. The central mechanism involves specific binding to the E3 ubiquitin ligase cereblon (CRBN), forming an E3 ubiquitin ligase complex that induces rapid ubiquitination and degradation of transcription factors Ikaros (IKZF1) and Aiolos (IKZF3). This process mediates therapeutic effects via several key pathways: Downregulation of BCR signaling through suppression of critical molecules (e.g., IRF4 and MYC), inhibiting tumor proliferation; Inhibition of NF-κB pathway activation, blocking pro-survival inflammatory cytokine effects in the tumor microenvironment; Upregulation of interferon signaling, enhancing tumor cell susceptibility to immune-mediated destruction. In addition, Len demonstrates synergistic efficacy when combined with RTX, primarily by augmenting ADCC through Fcγ receptor IIIa (CD16a) potentiation (59–61).

A multicenter, prospective phase II study (62) conducted by French Oculo-Cerebral lymphoma (LOC) Network and the Lymphoma Study Association (LYSA) aimed to evaluate the efficacy and safety of Len in combination with RTX (R2) for treating R/R PCNSL or PVRL. A total of 45 patients who were eligible for efficacy assessment were enrolled, with an ORR of 67% during the best induction period. At a median follow-up of 19.2 months, the median PFS and OS were 7.8 months and 17.7 months, respectively. Notably, no unexpected toxicities were observed throughout the treatment process. On the other hand, a multicenter, single-arm, open-label phase 2 study (63) by Yuan and colleagues explored the efficacy and safety of Len, RTX, and HD-MTX(R2-MTX) combinations for the initial treatment of PCNSL, followed by R2 maintenance therapy. The study included 17 patients, with the best ORR during induction treatment at 94.1%. At a median follow-up of 23 months, the projected 2-year PFS rate was 58.8% and the 3-year OS rate was 76.0%. All patients experienced TRAEs, but no patients discontinued therapy due to AEs.

Pomalidomide (POM) as a third-generation IMiD has a structure similar to Len but exhibits stronger anti-tumor activity with comparable safety (64, 65). Studies in rat models have shown that the cerebrospinal fluid (CSF) penetration rate of POM reaches 39% (66). In a phase I study of POM combined with dexamethasone treatment for R/R PCNSL/PVRL (67), 25 patients were enrolled, with an ORR of 48%. At a median follow-up of 16.5 months, the median PFS was 5.3 months, and the treatment was shown to be well tolerated.

In summary, the aforementioned research findings have revealed the potential of IMiDs in the treatment of IP-LBCLs, demonstrating good tolerance and high efficacy, thus providing clinicians with more treatment options.

#### 2.2.3 Immune checkpoint inhibitors

ICIs primarily enhance the cytotoxic activity of host T cells by blocking inhibitory signals of T cell activation, rather than directly targeting tumor cells. They work by stimulating the host immune system to exert anti-tumor effects (68). The application of these inhibitors in lymphoma primarily targets two key immune checkpoint proteins: programmed cell death protein 1 (PD-1) and programmed death ligand 1 (PD-L1). PD-1 is predominantly expressed on the surface of T cells, while PD-L1 is often expressed on the surface of tumor cells. Upon binding of PD-1 to PD-L1, the anti-tumor activity of T cells is suppressed. Therefore, PD-1/PD-L1 inhibitors block the interaction between PD-1 and PD-L1, thereby restoring the recognition and attack capabilities of T cells against tumor cell (69). Existing research indicates that increased PD-L1/PD-L2 gene copies or translocations are relatively common in patients with PCNSL and PTL (21, 23, 70). Additionally, there are case reports suggesting that the immune escape mechanism in IVLBCL may be associated with PD-L1 (71). However, some researchers point out that the immune escape mechanism in PTL is mainly related to the loss of HLA, rather than changes in PD-L1/PD-L2 (72).

Recent findings reported by researchers Nayak and colleagues have important implications (73). They observed 5 patients with R/R IP-LBCLs, including 4 with R/R PCNSL and 1 with CNS recurrence of PTL. All patients received nivolumab treatment, with the best ORR reaching 100% during the induction phase, and all patients had PFS exceeding 13 months. Moreover, researchers Terziev and colleagues (74) reported a case of a PCNSL patient who experienced a second recurrence and received three ASCT. Following the third ASCT, the patient received sequential nivolumab maintenance therapy for 1 year, achieving CR and maintaining it for over 24 months. In a phase II study (75) led by Zeng et al., the combination of sintilimab with HD-MTX, temozolomide, and RTX (SMTR) was used for induction therapy in patients with newly diagnosed PCNSL. This regimen demonstrated an extremely high ORR, reaching 96.3%, with a CRR of 92.6%. At a median follow-up of 24.4 months, the 2-year PFS and OS rates were 57.2% and 91.5%, respectively. The most common grade 3 AE was increased alanine aminotransferase levels (17.9%), but these patients with elevated transaminase levels could be managed reversibly with liver protection treatments. None of the patients reduced or ceased treatment due to drug toxicity.

These groundbreaking findings undoubtedly illuminate novel therapeutic avenues and renewed hope for patients with IP-LBCLs. ICIs such as nivolumab and sintilimab have demonstrated substantial clinical efficacy and favorable safety profiles in IP-LBCLs, representing a paradigm shift in treatment algorithms. However, emerging evidence necessitates cautious optimism, as ICIs may precipitate immune-related AEs (irAEs) including pneumonitis, hepatitis, colitis, endocrine dysregulation (e.g., hypothyroidism), and neurotoxicity (e.g., encephalitis) (76). A seminal retrospective analysis by Fonseca et al. (77, 78) of 64 patients developing neurological irAEs post-ICIs therapy revealed alarming outcomes: mortality rates reaching 10-29%, with 40-50% of survivors acquiring persistent severe disability. These findings are corroborated by Farina et al. (79), who documented particularly dismal prognoses for ICI-induced focal encephalitis. While current studies of ICIs in PCNSL cohorts report no CNS-specific toxicity signals, clinicians must maintain heightened vigilance.

## 3 Novel treatment strategies

### 3.1 Targeted therapy

#### 3.1.1 Phosphoinositide 3-kinase/mammalian target of rapamycin inhibitors

PI3K is an intracellular lipid phosphatidylinositol kinase that converts phosphatidylinositol 4,5-bisphosphate (PIP2) to phosphatidylinositol 3,4,5-trisphosphate (PIP3) by specifically catalyzing the phosphorylation of the hydroxyl at the third position of phosphatidylinositol. The production of PIP3 facilitates the recruitment and activation of AKT (80). Post-activation, AKT acts as a signaling molecule, further activating downstream signaling pathways and initiating a cascade of signaling reactions. The PI3K/AKT/mTOR signaling pathway plays a critical role in the regulation of cell growth and proliferation by integrating signals from growth factors, hormones, nutrients, and energy metabolism, thereby regulating protein synthesis and influencing cell survival and proliferation (80, 81). Recent studies have shown that there is abnormal activation of the PI3K/AKT/mTOR signaling pathway in PCNSL patients, and this phenomenon is closely associated with poor prognosis (82). Therefore, exploring targeted drugs against the PI3K/AKT/mTOR signaling pathway, as well as integrating these drugs with other therapeutic strategies, is of great significance for improving the prognosis of IP-LBCL patients.

A multicentric phase II clinical trial, which is part of targeted therapies, investigated the use of the mTOR inhibitor temsirolimus in the treatment of R/R PCNSL (83). The study included 37 patients, with results showing an optimal ORR of 54%, a median PFS of 2.1 months, and an OS of 3.7 months. 56.8% of patients experienced grade ≥3 AEs. TRAEs led to death in 5 patients (13%), including 2 cases of pneumonia, and 1 case each of gastrointestinal infection with sepsis, sepsis, and cerebral hemorrhage. Additionally, 5 patients succumbed to disease progression. In another prospective study (84), the efficacy of the PI3K inhibitor Buparlisib (BKM120) as a monotherapy for R/R CNSL was evaluated. Among the four enrolled patients, the ORR was only 25%, with a median PFS and OS of 39 days and 196 days, respectively. One patient had to discontinue treatment due to psychiatric symptoms. In light of the low efficacy of this study, the trial was suspended. However, in a preclinical study (85), researchers established an orthotopic CNSL model by intracranially injecting A20-Luciferase-GFP cells into SCID mice. The therapeutic efficacy of BEBT-908, a novel PI3K/HDAC dual inhibitor, was subsequently evaluated through intravenous administration. The experiment demonstrated that BEBT-908 exhibited excellent BBB permeability in the intracranial lymphoma model and effectively inhibited tumor growth, extending the survival of host mice. Mechanistically, BEBT-908 downregulated C-MYC expression, induced iron death, and ultimately led to tumor shrinkage. Based on these positive findings, we anticipate that BEBT-908 may serve as a therapeutic option for IP-LBCLs in the future.

Current applications of PI3K/mTOR inhibitors in IP-LBCLs have not demonstrated long-term PFS or OS benefits, while long-term administration is associated with severe TRAEs. However, their potential in combination therapies remains promising for enhancing therapeutic efficacy and safety profiles.

#### 3.1.2 Histone deacetylase inhibitors

Histone acetyltransferases (HATs) and HDACs dynamically regulate the epigenetic landscape by modulating acetylation post-translational modifications (PTMs) of histones and non-histone substrates. HATs catalyze the transfer of acetyl groups to specific lysine residues on histones and functional proteins (e.g., transcription factors, metabolic enzymes), whereas HDACs remove these acetyl marks. The equilibrium between these opposing activities governs chromatin architecture and transcriptional fidelity. Dysregulation of this balance disrupts cell cycle control, metabolic reprogramming, and epigenetic plasticity, ultimately driving oncogenesis (86, 87).

HDAC inhibitors such as chidamide restore HAT/HDAC-mediated acetylation homeostasis by selectively inhibiting HDAC activity. This reactivates tumor suppressor genes, suppresses pro-survival signaling pathways, and remodels the tumor immune microenvironment (88, 89). Chidamide has demonstrated marked efficacy in hematologic malignancies, particularly in combination therapies. Recent studies highlight its ability to improve outcomes in systemic DLBCL with high-risk molecular features (90–92).

Moreover, chidamide’s unique BBB penetrative capacity positions it as a breakthrough candidate for PCNSL (93). A phase 2 trial by Chen et al. evaluated the CR-MTX regimen (chidamide + RTX + HD-MTX) in newly diagnosed PCNSL (94). Among 9 evaluable patients, the ORR post-induction reached 89% (CRR 78%), with median PFS of 13 months. No grade ≥3 TRAEs were observed. These findings underscore the promise of epigenetic-based combinatorial strategies for IP-LBCLs.

#### 3.1.3 Nuclear export protein 1 inhibitors

XPO1 is a primary nuclear export protein in cells, primarily responsible for the export of molecules such as proteins and various types of RNA from the nucleus to the cytoplasm (95). Nonetheless, overexpression of XPO1 is a common phenomenon in multiple malignant tumors, leading to uneven distribution of regulatory proteins within the cell and uncontrolled proliferation of tumor cells, thus facilitating tumor progression. Consequently, targeted therapeutic strategies against XPO1 have emerged as a new direction in the field of tumor therapy. Selinexor, as a small molecule inhibitor, can selectively inhibit the activity of XPO1, thus blocking its mediating nuclear export process. This inhibition leads to the accumulation of tumor suppressor proteins and cell cycle regulatory factors within the nucleus, which ultimately induces cell cycle arrest and demonstrates specific antitumor activity (96). In June 2020, the FDA approved selinexor for the treatment of R/R DLBCL, marking a significant advancement in its clinical application (97). It is noteworthy that selinexor possesses the ability to penetrate the BBB. Clinical case reports (98) indicate that selinexor has significant efficacy in inhibiting CNS involvement in R/R DLBCL. This provides strong evidence supporting the use of selinexor as a novel targeted therapeutic agent for IP-LBCLs.

#### 3.1.4 B-cell lymphoma-2 inhibitors

The BCL-2 family plays a crucial role in regulating the intrinsic apoptosis pathway. The BCL-2 protein belongs to a group of anti-apoptosis proteins, and its abnormal expression is closely associated with the occurrence and development of tumors (99). Therefore, specific inhibitors targeting the BCL-2 protein have become one of the key strategies for promoting apoptosis and achieving anti-tumor effects. A recent retrospective study using immunohistochemical technology has found that approximately 60% of patients with PCNSL have overexpression of BCL-2 (100). This finding provides strong clinical evidence for targeted therapy against BCL-2. In SICD mouse models, BCL2 inhibitors (venetoclax or navitoclax) in combination with immunochemotherapy regimens have shown significant synergistic effects, to some extent inhibiting the occurrence of CNSL (101). Although no clinical reports of BCL-2 inhibitors in IP-LBCLs exist to date, a phase Ib clinical trial (102) presented at the 2024 American Society of Hematology (ASH) Annual Meeting investigated the BCL-2 inhibitor venetoclax combined with Pola-R-CHP in treatment-naïve DLBCL patients with high BCL-2 expression (immunohistochemical positivity ≥50%). The study enrolled 50 patients, achieving a CRR exceeding 80%. With a median follow-up of 15.5 months, the 1-year PFS and OS rates were 75.5% and 91.7%, respectively.

These research findings provide important experimental evidence for the clinical application of BCL-2 inhibitors and also offer new options for clinicians treating IP-LBCLs.

#### 3.1.5 Interleukin-1 receptor-associated kinase 4 inhibitors

IRAK4, a pivotal kinase in the TLR/MYD88-dependent signaling pathway, is critically implicated in the pathogenesis and progression of various hematologic malignancies, including DLBCL and acute myeloid leukemia (103, 104). When MYD88 mutations occur, the sustained activation of IRAK4 can lead to abnormal activation of the NF-κB signaling pathway, thereby promoting cell proliferation (105). Emavusertib (CA-4948) acts as a selective IRAK4 inhibitor and has garnered extensive attention due to its unique mechanism of action and favorable pharmacological properties. Particularly, when used in combination with ibrutinib, emavusertib can significantly increase the number of cells undergoing apoptosis, displaying a potential synergistic antitumor effect (106). Additionally, one notable advantage of emavusertib is its ability to effectively cross the BBB, which is of great significance for the treatment of hematological diseases involving the CNS (107). In a study evaluating the efficacy and safety of emavusertib in combination with ibrutinib for R/R PCNSL (108), an ORR of 50% was observed in 12 assessable patients. At the same time, there have been no reports of fatal TRAEs, and most AEs of ≥ grade 3 are reversible and manageable, indicating good safety characteristics. In summary, emavusertib holds promising prospects for the treatment of IP-LBCLs, and its precise targeting of IRAK4 provides a new therapeutic approach for hematological diseases.

### 3.2 Immunotherapy

#### 3.2.1 Bispecific antibodies

Glofitamab, as an innovative 2:1 structured IgG1-type fully humanized bispecific antibody, has demonstrated significant anti-tumor potential. The antibody possesses two CD20 domains and one CD3 domain, with a unique 2:1 structure that not only enhances the targeting of lymphomas expressing CD20 positivity but also effectively exerts an anti-tumor effect by guiding CD3 effector T cells to the surface or surrounding area of tumor cells (109). In the pivotal NP30180 phase II study (110), glofitamab showed a promising therapeutic effect in 155 patients with R/R DLBCL. The study results indicate that glofitamab’s ORR was 52%, with a CRR of 39%. This achievement not only opens a new chapter in the treatment of R/R DLBCL with bispecific antibodies but also provides new insights and options for clinical treatment of other B-cell lymphomas.

Godfrey et al. conducted the first analysis of glofitamab in the treatment of R/R CNSL (111). In this study, a total of 4 patients were included, and CSF samples were collected from this subset of patients. The results showed that glofitamab was detected in the CSF of all 4 patients, with concentrations ranging from 0.00632 to 0.0296 μg/ml. Notably, 3 of the 4 patients experienced relief after treatment with glofitamab. During the treatment, only 2 patients developed a transient grade 1-2 cytokine release syndrome (CRS), indicating that glofitamab exhibits good efficacy and safety in the treatment of R/R CNSL.

#### 3.2.2 Chimeric antigen receptor T cell immunotherapy

CAR-T immunotherapy represents a revolutionary strategy in tumor treatment. This therapy involves genetically engineering a patient’s own T cells to enable them to specifically recognize and attack tumor cells. Currently, CAR-T cell therapy has demonstrated significant efficacy in the treatment of R/R DLBCL (112). Nevertheless, this therapy is also associated with some unique toxicities, including neurotoxicity. It is noteworthy that CAR-T cells can enter the CNS even in lymphoma patients without pre-existing CNS involvement, leading to immune effector cell associated neurotoxicity syndrome(ICANS) (113). For these reasons, due to concerns about the onset and progression of ICANS, research on the use of CAR-T therapy in PCNSL is mostly limited to small-scale retrospective and prospective studies.

The study by Mercadal and colleagues (114) is a clinical trial investigating the use of CD19 CAR-T cell therapy for R/R PCNSL. The study enrolled 24 patients with R/R PCNSL, of whom 23 were evaluable for response. By day 100, the ORR reached 61%. The median follow-up after CAR-T infusion was 26 months, with a 2-year PFS rate of 28% and an OS rate of 50%. A phase I/II clinical trial led by Frigault et al. (115) evaluated CD19 CAR-T cell therapy in 12 patients with R/R PCNSL, with a median follow-up of 12.2 months. The study demonstrated a best ORR of 58.3% and CRR of 50.0%, with median response duration of 3.1 months. Safety analysis revealed grade 1 CRS in 58.3% (7/12) and grade ≤2 ICANS in 41.6% (5/12), with only 1 case of grade 3 ICANS. No treatment-related mortality was observed.

Another retrospective study conducted within the French LOC network included 25 patients with R/R PCNSL who had received CAR-T treatment (116). This study reported a best ORR of 80%, with a median follow-up of 19.4 months after CAR-T cell infusion, leading to a median PFS of 8.4 months and a median OS of 21.2 months. Two patients (8%) experienced ≥ grade 3 CRS, and five patients (20%) developed ≥ grade 3 ICANS. A meta-analysis evaluating CAR-T therapy in R/R CNSL revealed distinct toxicity profiles, with 30 PCNSL cases. Any-grade CRS and ICANS occurred in 70% and 53% of patients respectively, with grade 3-4 CRS and ICANS observed in 13% and 18% of cases. These toxicity profiles align with CAR-T registrational trials in systemic LBCL, without elevated neurotoxicity signals (117).

The collective evidence of high response rates and manageable toxicity supports CAR-T therapy as a promising therapeutic option for IP-LBCLs. Ongoing research should optimize durability and refine toxicity mitigation strategies.

## 4 Limitations

While this review provides a comprehensive analysis of recent advances in targeted and immunotherapeutic approaches for IP-LBCLs, several limitations warrant consideration. First, given the rarity of IP-LBCLs and consequent limited patient population, conducting large-scale clinical trials remains challenging. Consequently, current evidence predominantly originates from case reports or small cohorts, inherently increasing the risk of stochastic bias and reducing statistical power. Therefore, cautious interpretation is warranted to avoid overgeneralization of findings. Second, inherent language bias must be acknowledged, as our inclusion criteria prioritized English-language publications. This selective approach may not encompass all relevant studies reported in other languages, potentially introducing geographic or institutional reporting biases.

## 5 Summary

**Figure 2:**
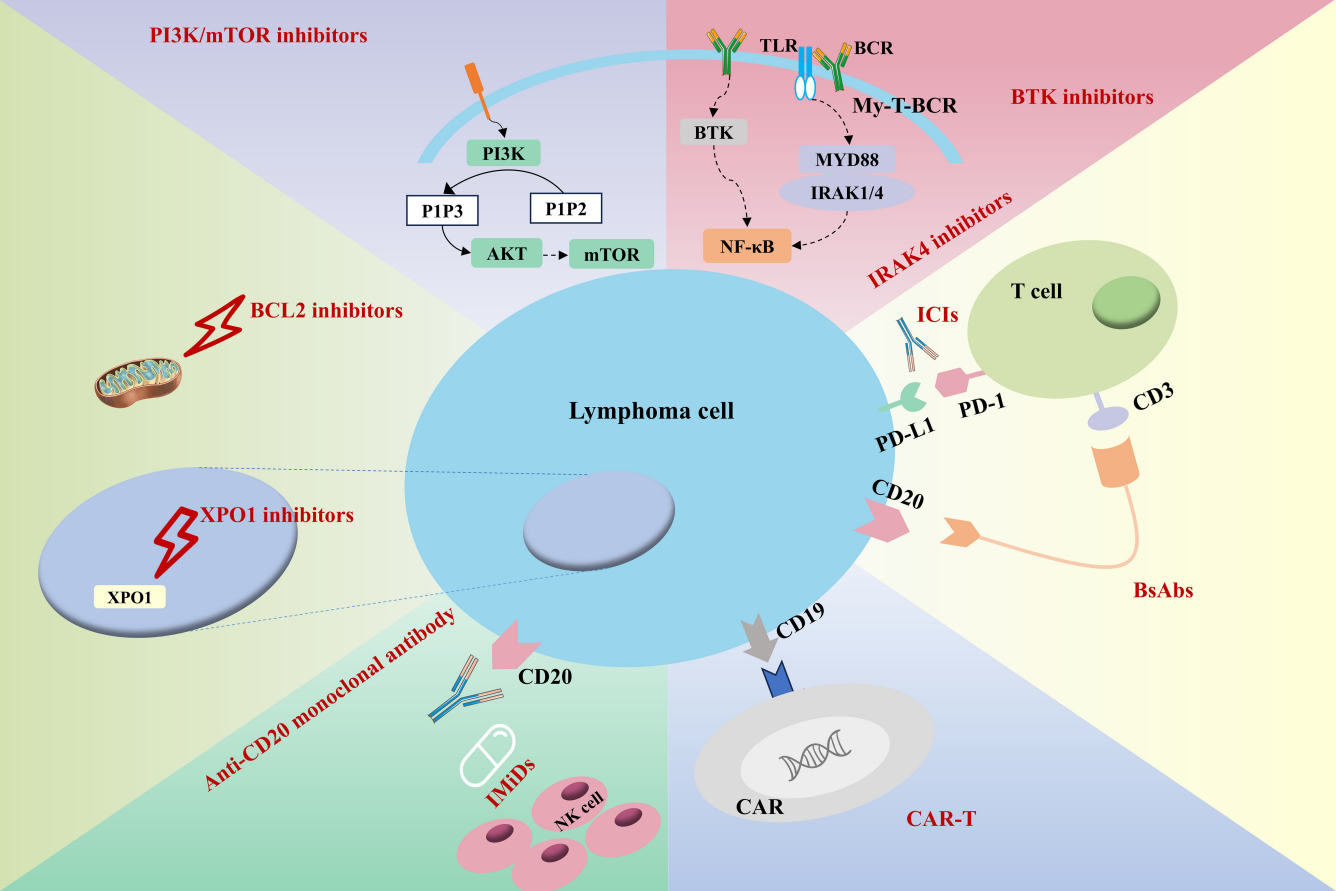
Mechanisms of different treatment regimens. (BTKi, Bruton’s tyrosine kinase inhibitor; IRAK4, Interleukin-1 receptor-associated kinase 4; ICIs, Immune checkpoint inhibitors; BsAbs, Bispecific antibodies; CAR, chimeric antigen receptor).

In the era of targeted and immunotherapies, therapeutic strategies for IP-LBCLs have expanded significantly (**Figure 2**, **Table 2**). Agents including BTK inhibitors, IMiDs, and ICIs have demonstrated substantial clinical efficacy. Given that most IP-LBCLs belong to the molecularly defined MCD subtype with shared biological features, BTK inhibitors may represent an optimal therapeutic backbone for this lymphoma entity.

**Table 2:** Different immunotherapy regimens for the treatment of IP-LBCLs.

		Regimen	Disease status	Patients, n	Effectiveness	AEs (Grade ≥3)	Reference
Current treatments	BTKi	Ibrutinib	R/R PCNSL	13	ORR: 77%; median PFS and OS: 4.6 and 15 months		(32)
		Ibrutinib	R/R PCNSL	31	ORR: 74%; median PFS: 4.5 months		(33)
		MIT	TN PCNSL	33	ORR: 94%; 1-year PFS: 66.4%	Neutropenia: 9%; Thrombocytopenia: 6%; Subdural hematoma: 6%; Atrial fibrillation: 3%	(34)
		Ibrutinib	TN PCNSL	20	2-year PFS and OS: 72.6% and 89%	Neutropenia: 20%; High blood pressure: 20%	(36)
		ZA	R/R PCNSL	34	ORR: 64.7%; median PFS: 4.9 months	Thrombocytopenia: 47.1%; Neutropenia: 22.6%; Pneumonia: 11.8%	(118)
		R-MTO	TN PCNSL	19	ORR: 94.73%		(37)
		Tirabrutinib	TN PCNSL	44	ORR: 64%; median PFS: 2.9 months	Neutropenia: 9.1%; Lymphopenia, leukopenia, and erythema multiforme: 6.8% each	(38)
		ZR-MTX	TN PCNSL	15	ORR: 66.7%; 2-year PFS and OS: 38.9% and 67.5%	Neutropenia: 20%	(35)
		HZ-A-018	R/R CNSL	26	ORR:50.0%	Neutropenia: 11.5%	(39)
		ZR-CHOP	TN IVLBCL	9	ORR: 100%	Neutropenia: 66.7%; Anemia: 22.2%; Thrombocytopenia: 22.2%	(41)
	Rituximab	R-MBVP	TN PCNSL	99	1-year EFS:52%; 2-year OS: 71%	Infections: 21%; Haematological toxicity: 12%; Nervous system disorders: 12%; Died: 3%	(48)
		R-MA	TN PCNSL		ORR: 74%; 7-year OS: 37%	Neutropenia: 56%; Thrombocytopenia: 74%; Anemia: 47%	(50)
		MATrix	TN PCNSL		ORR: 87%; 7-year OS:56%	Neutropenia: 67%; Thrombocytopenia: 83%; Anemia: 36%	(50)
		R-CHOP	TN PTL	54	CRR: 98%; 5-year PFS and OS: 91% and 92%	Neutropenia: 29.6%;Febrile neutropenia: 11.1%	(55)
		R-CHOP	TN IVLBCL	37	CRR: 85%; 2-year PFS and OS: 76% and 92%	Leucocytopenia and neutropenia: 100%; Febrile neutropenia: 34%;	(56)
	IMiDs	R2	R/R PCNSL	45	ORR: 67%; median PFS and OS: 7.8 and 17.7 months	Neutropenia: 44%; Pneumonia: 6.7%	(62)
		R2-MTX	TN PCNSL	17	ORR: 88.2%; 2-year PFS: 58.8%; 3-year OS: 76.0%	Neutropenia: 47.1%; Leukopenia: 23.5%	(63)
		POM	R/R PCNSL	25	ORR: 48%; median PFS: 5.3 months	Neutropenia: 21%; Pneumonia: 12%	(67)
	ICIs	Nivolumab	R/R CNSL	1	CRR: 100%; PFS>24 months		(74)
		Nivolumab	R/R PCNSL/PTL	5	ORR: 100%; PFS>13 months	Renal insufficiency: 20%	(73)
		SMTR	TN PCNSL	27	ORR: 96.3%; 2-year PFS and OS: 57.2% and 91.5%	Increased levels of alanine aminotransferase (17.9%) and aspartate aminotransferase (14.3%)	(75)
Emerging therapies	PI3K/mTORi	Temsirolimus	R/R PCNSL	37	ORR: 54%; median PFS and OS: 2.1 and 3.7 months	Hyperglycemia: 29.7%; Thrombocytopenia: 21.6%; Infection: 19%; Anemia: 10.8%	(83)
		Buparlisib	R/R CNSL	4	ORR: 25%; median PFS and OS: 39 and 196 days		(84)
	HDACi	CR-MTX	TN PCNSL	9	ORR:89%; median PFS: 13 months	Neutropenia: 20%; Thrombocytopenia: 20%	(94)
	IRAK4i	Emavusertib and ibrutinib	R/R PCNSL	12	ORR: 50%		(108)
	BsAbs	Glofitamab	R/R CNSL	4	ORR: 75%		(111)
	CAR-T	Tisa-cel	R/R PCNSL	12	ORR: 58.3%	ICANS: 8.3%	(115)
		Axi-cel and tisa-cel	R/R PCNSL	24	ORR: 61%; 2-year PFS and OS: 28% and 50%	ICANS: 8.3%	(114)
		Axi-cel and tisa-cel	R/R PCNSL	25	ORR: 80%; median PFS and OS: 8.4 and 21.2 months	CRS: 8%; ICANS: 20%	(116)

IP-LBCLs, Primary Large B-cell Lymphomas of Immune-Privileged Sites; PCNSL, primary central nervous system lymphoma; PVRL, primary vitreoretinal lymphoma; PTL, primary testicular lymphoma; IVLBCL, intravascular large B-cell lymphoma; BTKi, Bruton’s tyrosine kinase inhibitor; MIT, methotrexate/ibrutinib/temozolomide; ZA, zanubrutinib/cytarabine; R-MTO, methotrexate/orelabrutinib/thiotepa/rituximab; ZR-MTX, zanubrutinib/rituximab/methotrexate; R-MBVP, rituximab/methotrexate/carmustine/teniposide/prednisone; R-MA, rituximab/methotrexate/cytarabine; MARix, methotrexate/cytarabine/rituximab/thioepa; R-CHOP, rituximab/cyclophosphamide/doxorubicin/vincristine/prednisone; IMiDs, immunomodulatory agents; R2, lenalidomide/rituximab; POM, pomalidomide; ICIs, Immune checkpoint inhibitors; SMTR, sintilimab/methotrexate/temozolomide/rituximab; CR-MTX, chidamide/rituximab/methotrexate; IRAK4i, Interleukin-1 receptor-associated kinase 4 inhibitors; HDACi, Histone deacetylase inhibitors; BsAbs, Bispecific antibodies; CAR, chimeric antigen receptor; Axi-cel, axicabtagene ciloleucel; tisa-cel, tisagenlecleucel; R/R, relapsed/refractory; TN, treat-naïve; CRS, cytokine release syndrome; ICANS, immune effector cell associated neurotoxicity syndrome; ORR, overall response rate; CR, complete response; EFS, event-free survival; PFS, progression-free survival; OS, overall survival.

Emerging modalities such as PI3K/mTOR inhibitors, HDAC inhibitors, XOP1 inhibitors, BCL-2 inhibitors, IRAK4 inhibitors, bispecific antibodies, and CAR-T therapies further diversify treatment options. Future research should focus on elucidating the mechanistic underpinnings of these interventions to optimize therapeutic indices and develop personalized approaches tailored to molecular vulnerabilities.
